# Impact of a reimbursement policy change on treatment with erenumab in migraine – a real-world experience from Germany

**DOI:** 10.1186/s10194-023-01682-2

**Published:** 2023-10-30

**Authors:** Ja Bin Hong, Kristin Sophie Lange, Mira Fitzek, Lucas Hendrik Overeem, Paul Triller, Anke Siebert, Uwe Reuter, Bianca Raffaelli

**Affiliations:** 1 Department of Neurology, Charité-Universitätsmedizin Berlin, Berlin, Germany; 2 Universitätsmedizin Greifswald, Greifswald, Germany; 3 Clinician Scientist Program, Berlin Institute of Health (BIH) at Charité, Berlin, Germany

**Keywords:** Migraine, Preventive treatment, Monoclonal antibodies, Calcitonin gene-related peptide, Erenumab, Insurance coverage, Health policy

## Abstract

**Background:**

Monoclonal antibodies (mAbs) targeting the Calcitonin Gene-Related Peptide (CGRP) pathway are safe and effective treatments for migraine prevention. However, the high cost of these novel therapies has led to reimbursement policies requiring patients to try multiple traditional preventives before access. In Germany, a recent change in insurance policy significantly expanded coverage for the CGRP receptor mAb erenumab, enabling migraine patients who failed just one prior prophylactic medication to receive this mAb. Here, we compare the clinical response to treatment with erenumab in migraine patients treated using the old and new coverage policy.

**Methods:**

In this retrospective cohort study, we included CGRP-mAb naïve patients with episodic or chronic migraine, who started erenumab at our headache center according to either the old or the new insurance policy and received at least 3 consecutive injections. Headache diaries and electronic documentation were used to evaluate reductions in monthly headache and migraine days (MHD and MMD) and ≥ 50% and ≥ 30% responder rates at month 3 (weeks 9–12) of treatment.

**Results:**

We included 146 patients who received erenumab according to the old policy and 63 patients that were treated using the new policy. At weeks 9–12 of treatment, 37.7% of the old policy group had a 50% or greater reduction in MHD, compared to 63.5% of the new policy group (*P* < 0.001). Mean reduction in MHD was 5.02 days (SD = 5.46) and 6.67 days (SD = 5.32, *P* = 0.045) in the old and new policy cohort, respectively. After propensity score matching, the marginal effect of the new policy on treatment outcome was 2.29 days (standard error, SE: 0.715, *P* = 0.001) more reduction in MHD, and 30.1% (SE: 10.6%, *P* = 0.005) increase in ≥ 50% response rate for MHD.

**Conclusions:**

Starting erenumab earlier in the course of migraine progression in a real-world setting may lead to a better response than starting after multiple failed prophylactic attempts. Continually gathering real-world evidence may help policymakers in deciding how readily to cover CGRP-targeted therapies in migraine prevention.

## Background

Migraine is a highly prevalent neurological disorder that causes significant disability over decades. Over one billion individuals suffer from migraine worldwide, often in what would have been their most economically productive years [1, 2]. Until recently, preventive medications that were used to reduce migraine attack frequency were often burdened with debilitating side effects and uncertain efficacy [3, 4].

With the advent of therapeutic agents directed against Calcitonin Gene-Related Peptide (CGRP) and its receptor (CGRP-R), clinicians treating migraine patients are finally able to offer specific, more effective and well-tolerated options for prevention of both episodic and chronic migraine [5–9].

For many novel therapies, an important factor that prohibits their broad use is their cost. Pharmacoeconomic considerations are especially relevant for the insurance provider when the disease at hand, as in the case of migraine, has a high prevalence, and requires long-term treatment. When establishing criteria for insurance coverage of expensive new treatments, it is justified to exercise caution, as their widespread coverage and utilization could potentially lead to overwhelming costs for both insurers and taxpayers.

In the case of CGRP-targeted therapies, most country policies require patients to undergo multiple attempts with traditional oral prophylactic drugs before coverage of CGRP-targeted therapies. However, the question arises whether it is indeed more cost-efficient to require patients to try multiple classes of prophylactic medications before they are allowed to receive a treatment that is potentially more effective and has significantly less side effects [4, 10]. This approach often results in delaying treatment with CGRP (-R) mAbs for several months, during which patients may endure significant side effects and further chronification of migraine, potentially reducing the efficacy of CGRP (-R) mAbs when they are finally prescribed [11].

In Germany, until October 2022, patients were required to have tried and failed or have contraindications to all prophylactic medication classes of first choice in order to have coverage for any CGRP (-R) mAbs. Failure to a preventive medication could be either due to lack of efficacy, or the occurrence of intolerable side effects that prohibited its use. Required prophylactic medications included at least one beta-blocker (either metoprolol, propranolol or bisoprolol), amitriptyline, flunarizine, topiramate, and onabotulinumtoxinA in case of chronic migraine. Recent changes in the requirements for insurance coverage of erenumab have made it possible for patients with at least four migraine days per month to receive treatment with erenumab after only one failed preventive medication attempt [12]. The decision to expand coverage for erenumab was based on the results of the HER-MES trial [10], where erenumab was compared directly with topiramate in the prevention of migraine through a randomized clinical trial, and showed a superior efficacy and tolerability profile. The monthly cost of therapy in Germany was 688,36€ for the 70 mg dose, and 1376.72€ for the 140 mg dose at the beginning of 2018 [13], and with subsequent price reductions 311.95€ (pharmacy retail price) for both the 70 mg and 140 mg dosages in 2022.

This change in coverage policy has provided us with an ideal natural setting to examine the impact of an earlier start of erenumab on patient outcomes. In this study, we sought to evaluate and compare the treatment response in patients that were treated with erenumab in our headache center according to the old vs. new coverage policies.

## Methods

### Study design and patient selection

We performed a single-center retrospective cohort study at the tertiary headache center at Charité-Universitätsmedizin Berlin, Germany. We screened all consecutive patients with the diagnosis of episodic or chronic migraine according to the ICHD-3 criteria [14], with or without aura, who were given a first injection of erenumab 70 or 140 mg between November 2018 and February 2023 at our headache center. Those who had been CGRP (-R) mAb-naïve before receiving the first dose of erenumab, and continued treatment with erenumab for at least three months were included in our study. We excluded patients that had been treated with a CGRP (-R) mAb before receiving erenumab, patients who participated in randomized clinical trials of CGRP (-R) mAbs, and patients with insufficient headache calendar data, defined as missing monthly headache days (MHD), either from headache calendars or from clinician’s notes, for the third month of treatment. Patients with a concomitant headache disorder other than tension-type headache in case of episodic migraine were also excluded. We compared patients treated after fulfillment of previous coverage requirements, i.e. those who tried and failed or had contraindications to all prophylactic medication classes of first choice (old policy) with patients that received erenumab according to the new treatment guideline, after at least one prophylactic medication trial (new policy). Patients who received erenumab after the policy change was introduced but fulfilled previous coverage requirements were grouped into the old policy cohort.

### Data collection and study outcomes

Baseline characteristics including basic demographic data, migraine history including previous prophylactic medication attempts, and migraine characteristics were collected from electronic medical records. Standardized headache calendars were primarily used to determine treatment response. Headache calendars were evaluated for the period of four weeks before the first erenumab injection (baseline period), and for each four-week interval before each subsequent erenumab injection. We recorded the number of MHD and monthly migraine days (MMD) of each four-week interval. Migraine days were defined as any headache day where the headache fulfilled the ICHD-3 [14] diagnostic criteria for migraine or an acute medication with triptans was used. If headache calendars were not available, MHD and MMD documented in the electronic clinician’s notes were used instead.

The primary outcome of interest was a ≥ 50% reduction in MHD at weeks 9–12 (3^rd^ month) of therapy. Secondary outcomes were a ≥ 30% reduction in MHD, a ≥ 50% and ≥ 30% reduction in MMD at weeks 9–12 and absolute change in MHD and MMD from baseline to weeks 9–12 of therapy.

### Statistical methods

Baseline demographic and clinical variables were summarized as mean and standard deviation (SD) for normally distributed numerical variables, median and interquartile range (IQR) for not normally distributed numerical variables or *n* (%) for categorical variables. Categorical outcomes (50% or 30% response) were summarized as *n* (%) and change in MMD and MHD as mean (SD). Normality was assessed graphically using Q-Q plots. The chi-square test of homogeneity was used to test for differences in dichotomous baseline variables and response rates between the old and new policy groups. The independent T-test was used to test for differences numerical baseline variables judged to be normally distributed, and differences in change in MHD and MMD from baseline between the old and new policy groups. The Mann–Whitney U test was used to compare differences in numerical baseline variables that were not normally distributed between the two groups.

Univariable and multivariable binomial logistic regression was used to investigate associations between clinical variables and ≥ 50% response in MHD. Linearity between continuous independent variables and the logit of the dependent variable was evaluated using the Box-Tidwell procedure [15]. We assessed for multicollinearity through an inspection of correlation coefficients and Tolerance/VIF values and checked the presence of outliers using standardized residuals. Variables entered into the model were sex, age, chronic migraine, disease duration, presence of daily headaches, MHD at baseline, comorbid depression, starting dose of 140 mg, in addition to the old vs. new policy group.

We used propensity score matching to account for confounding by covariates that differed between the two patient groups. Variables that differed between the two groups, excluding those that were a direct result of policy implementation, were included in the model used to estimate the propensity score (age, MHD at baseline, disease duration, chronic vs. episodic migraine, comorbid depression). We used optimal full matching [16] on the propensity score estimated using probit regression of patient group on covariates [17]. R [18], Rstudio [19] with the MatchIt [20] package were used to create the matched sample. The marginal effect of the policy change (average treatment effect of the treated, ATT) on response rate to therapy with erenumab was then estimated using a logistic regression model for the 50% response rate and with linear regression model for change in MHD, that included cohort category (old vs. new policy), age, sex, disease duration, MHD at baseline, the presence of daily headaches, and chronic vs. episodic migraine as independent variables. The marginal effects package was used to compute the estimated ATT [21].

## Results

**Fig. 1:**
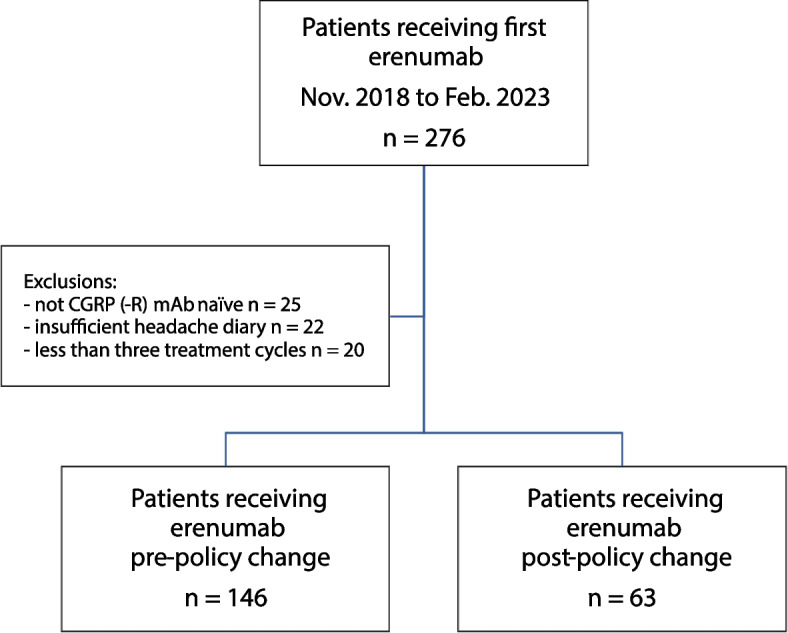
Flow-chart of included patients. CGRP (-R) mAb: Calcitonin gene-related peptide (-receptor) monoclonal antibody

Between November 2018 and February 2023, a total of 276 patients received their first injection of erenumab at our headache center. After eligibility assessment, we included 146 patients that had received erenumab after fulfilling previous requirements for coverage of CGRP (-R) mAbs (old policy), and 63 patients that had received erenumab according to new reimbursement requirements (new policy, Fig. 1).

Patients that began erenumab after meeting new reimbursement requirements were younger, had fewer MHDs at baseline and a shorter duration of disease before starting treatment with erenumab compared with the old policy cohort (Table 1). In addition, patients in the new policy cohort were more often given the 140 mg dose at the beginning (Table 1).

**Table 1:** Baseline characteristics by treatment group

Characteristic	Treatment group			All
	Old policy (*n* = 146)	New policy (*n* = 63)	*P*	(*n* = 209)
Age, mean (SD), yr	49.20 (11.73)	41.94 (12.03)	< 0.001	47.01 (12.26)
Sex (female), no. (%)	122 (83.6%)	52 (82.5%)	0.856	174 (83.3%)
Baseline MHD, median (IQR) d/mo	15 (11–21)	12 (9–16)	0.011	14 (10–20)
Baseline MMD, median (IQR) d/mo	13 (9–16)	11 (7–13)	0.011	12 (9–15)
Number of previous preventive medications, median (IQR)	5 (4–6)	2 (1–2)	< 0.001	4 (2–5)
Medication overuse, no. (%)	72 (49.3%)	18 (28.5%)	0.356	90 (43.1%)
Chronic migraine, no. (%)	94 (64.4%)	23 (36.5%)	< 0.001	117 (56.0%)
Disease duration, mean (SD), yr	30.52 (12.41)	19.60 (12.20)	< 0.001	27.22 (13.03)
Comorbid depression, no. (%)	51 (34.9%)	14 (22.2%)	0.069	65 (31.1%)
Full starting dose (140 mg), no. (%)	10 (6.8%)	32 (50.8%)	< 0.001	42 (20.1%)

*Abbreviations*: *MHD* monthly headache days, *MMD* monthly migraine days, *SD* standard deviation, *IQR* interquartile range

**Fig. 2:**
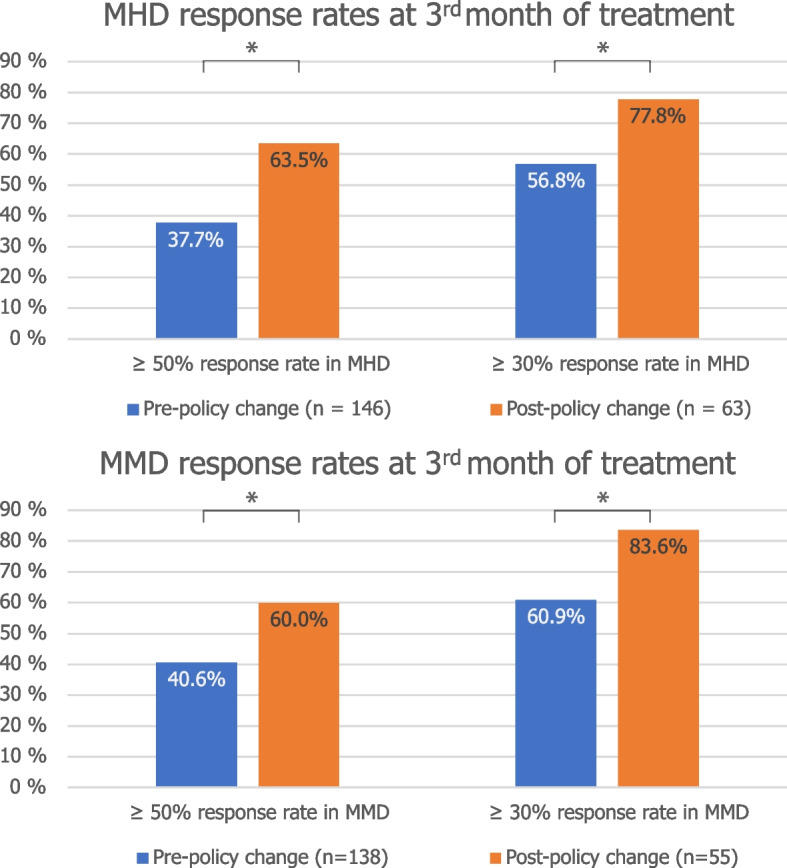
≥ 50% and ≥ 30% response rates in MHD **a** and MMD **b** at third month of treatment. The asterisk (*) is used to indicate *P* < 0.05. MHD: monthly headache days, MMD: monthly migraine days

At weeks 9–12 of treatment, 55 (37.7%) of the 163 patients in the old policy cohort and 40 (63.5%) patients of the 63 patients in the new policy cohort had a 50% or greater reduction in MHD compared to baseline (*P* < 0.001). Response rates at weeks 9–12 for the threshold of ≥ 30% reduction in MHD were 56.8% and 77.8% for the old and new policy cohorts respectively (*P* = 0.004, Table 2, Fig. 2).

**Table 2:** Efficacy outcomes at weeks 9–12 of treatment

	Treatment group		*P*	All
	Old policy (*n* = 146)	New policy (*n* = 63)		(*n* = 209)
≥ 50% response rate in MHD, no. (%)	55 (37.7%)	40 (63.5%)	< 0.001	95 (45.5%)
≥ 30% response rate in MHD, no. (%)	83 (56.8%)	49 (77.8%)	0.004	132 (63.2%)
Change in MHD at 3 months, mean (SD)	-5.02 (5.46)	-6.67 (5.32)	0.045	-5.52 (5.45)
	Old policy (*n* = 138)	New policy (*n* = 55)		All (*n* = 193)
≥ 50% response rate in MMD, no. (%)	56 (40.6%)	33 (60.0%)	0.015	89 (46.1%)
≥ 30% response rate in MMD, no. (%)	84 (60.9%)	46 (83.6%)	0.002	130 (67.4%)
Change in MMD at 3 months, mean (SD)	-4.78 (5.02)	-6.12 (5.04)	0.095	-5.17 (5.05)

*Abbreviations*: *MHD* monthly headache days, *MMD* monthly migraine days, *SD* standard deviation

**Fig. 3:**
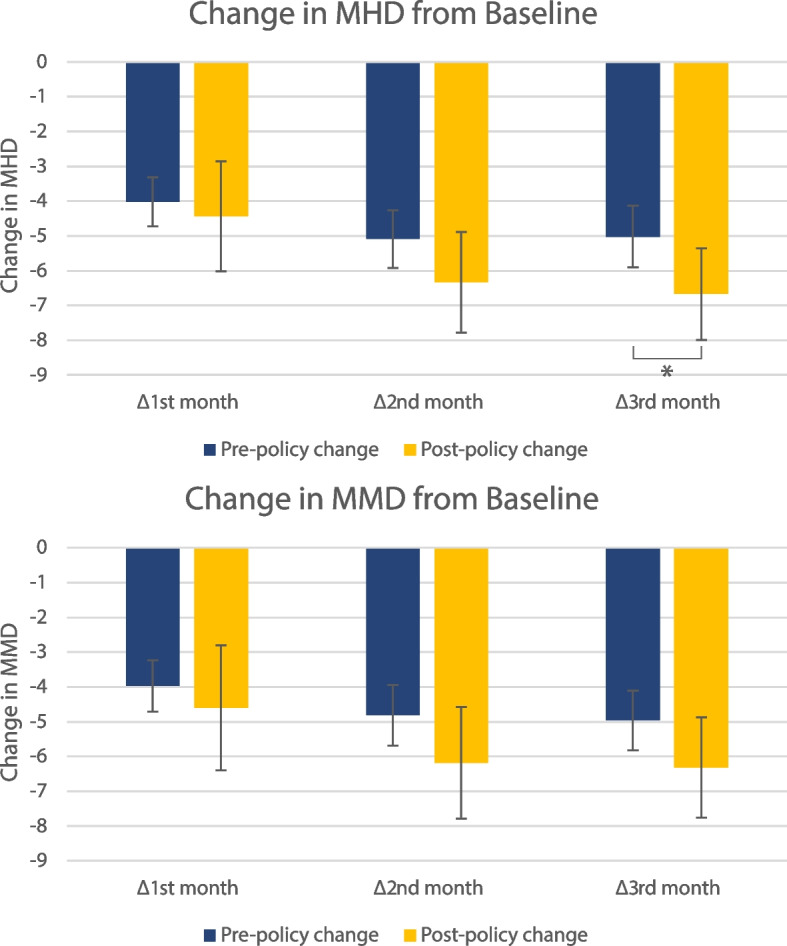
Change in MHD **a** and MMD **b** from baseline at first, second and third month of treatment. The asterisk (*) is used to indicate *P* < 0.05. MHD: monthly headache days, MMD: monthly migraine days. Error bars represent 95% confidence intervals

A ≥ 50% reduction in MMD at weeks 9–12 was achieved by 40.6% of patients in the old policy cohort, and by 60.0% of patients in the new policy cohort (*P* = 0.015). Response rates for ≥ 30% reduction in MMD were 60.9% and 83.6% for the old and new policy cohorts respectively (*P* = 0.002, Table 2, Fig. 2). Absolute changes in MHD and MMD from baseline to week 9–12 are reported in Table 2 and Fig. 3. We observed a greater difference in the absolute reductions in MHD and MMD between the old and new policy cohort with increasing duration of therapy (mean difference in reductions of MHD and MMD between old and new policy cohorts of 1.65 and 1.35 at month 3 vs. 0.42 and 0.63 days at month 1, Fig. 3).

We first performed univariable logistic regression analyses for each baseline variable that differed between the old and new policy groups (treatment group, sex, age, duration of disease, MHD at baseline, the presence of daily headaches, starting dose of erenumab, comorbid depression and episodic vs. chronic migraine) except for the number of prior preventive medications, the results of which are displayed in Table 3. We then performed a multivariable logistic regression for the outcome of a ≥ 50% response in MHD, with treatment group, sex, age, duration of disease, MHD at baseline, the presence of daily headaches, starting dose of erenumab, comorbid depression and episodic vs. chronic migraine as covariates, displayed in Table 4. Results of the multivariable analysis showed that being treated according to the new reimbursement policy led to a higher likelihood of responding with ≥ 50% reduction in MHD (OR = 2.44, 95% CI: 1.10–5.39, *P* = 0.03, Table 4) independent of baseline variables included as covariates.

**Table 3:** Results of univariable logistic regression predicting likelihood of ≥ 50% response in MHD

	Coefficient (β)	S.E	Wald	df	*P*	Odds Ratio	95% CI for Odds Ratio	
							Lower	Upper
New policy	1.06	0.31	11.44	1	0.01	2.88	1.56	5.30
Sex (female)	-0.43	0.37	1.31	1	0.25	0.65	0.32	1.35
Age (yrs)	-0.01	0.11	0.43	1	0.51	0.99	0.97	1.02
Disease duration (yrs)	-0.01	0.11	0.95	1	0.33	0.99	0.97	1.01
MHD at baseline	-0.52	0.02	5.87	1	0.02	0.95	0.91	0.99
Daily headaches	-0.58	0.43	1.80	1	0.18	0.56	0.24	1.31
Start with 140 mg	0.71	0.35	4.11	1	0.04	2.04	1.02	4.06
Comorbid depression	-0.60	0.31	3.81	1	0.05	0.55	0.30	1.00
Chronic migraine	-0.64	0.28	5.20	1	0.23	0.53	0.30	0.91

*Abbreviations*: *MHD* monthly headache days *S.E.* standard error *CI* confidence interval

**Table 4:** Results of multivariable logistic regression predicting likelihood of ≥ 50% response in MHD

	Coefficient (β)	S.E	Wald	df	*P*	Odds Ratio	95% CI for Odds Ratio	
							Lower	Upper
New policy	0.89	0.40	4.86	1	0.03	2.44	1.10	5.39
Sex (female)	-0.51	0.41	1.58	1	0.21	0.60	0.27	1.33
Age (yrs)	-0.01	0.02	0.16	1	0.69	0.99	0.96	1.03
Disease duration (yrs)	0.01	0.02	0.26	1	0.61	1.01	0.98	1.04
MHD at baseline	-0.44	0.04	1.52	1	0.22	0.96	0.89	1.03
Daily headaches	0.06	0.67	0.01	1	0.93	1.06	0.29	3.91
Start with 140 mg	0.08	0.44	0.04	1	0.85	1.09	0.46	2.57
Comorbid depression	-0.53	0.33	2.65	1	0.10	0.59	0.31	1.12
Chronic migraine	-0.13	0.35	0.14	1	0.71	0.88	0.45	1.73
Constant	0.93	0.90	1.07	1	0.30	2.54		

Variables included in the model: old vs. new policy, sex, age, disease duration, MHD (monthly headache days) at baseline, the presence of daily headaches, erenumab starting dose of 140 mg, comorbid depression, and chronic migraine

*Abbreviations*: *MHD* monthly headache days *S.E.* standard error *CI* confidence interval

**Fig. 4:**
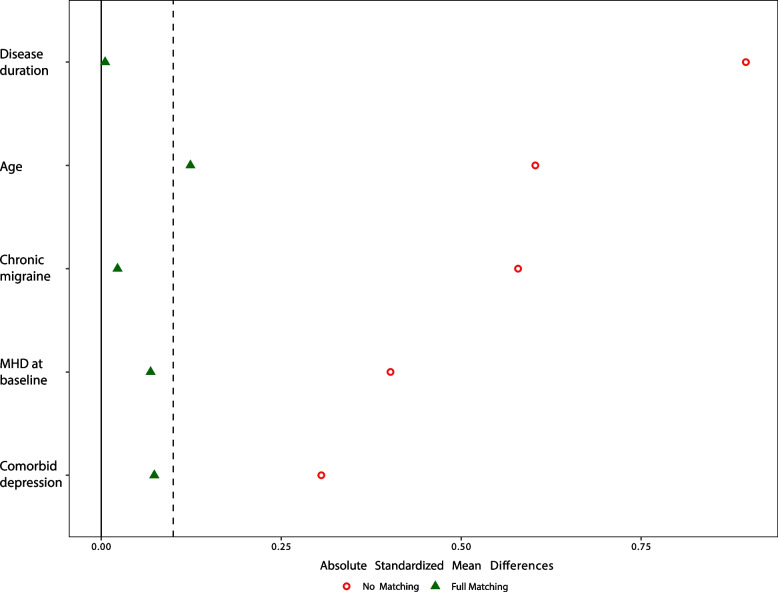
Absolute standardized mean differences for baseline variables before and after propensity score matching. MHD: monthly headache days. Broken line marks a standardized mean difference of 0.1

Because of the differences in baseline variables between the old and new policy groups, we performed propensity score matching to select cases from the old policy group that resembled the subjects in the new policy cohort. Standardized mean differences (SMD) reflecting covariate balance before and after full matching are displayed in Fig. 4. For all variables, the SMD was below or close to 0.1 after matching, which is generally considered acceptable [22]. The estimated average treatment effect of the policy change calculated after full matching was -2.29 days (SE: 0.715, *P* = 0.001) for change in MHD, and + 30.1% (SE: 10.6%, *P* = 0.005) for the 50% response rate for MHD.

## Discussion

This real-world study compared the treatment response to erenumab in migraine prevention before and after implementation of a new policy for insurance coverage in Germany. We observed that migraine patients who received erenumab according to the new policy, i.e., after a minimum of only one prior preventive treatment, had a higher rate of 50% response in MHD and MMD, and a higher absolute reduction in MHD than patients who had previously tried all first-line prophylactic medication classes. This difference was present even after adjusting for baseline variables that differed between the two groups using multivariable regression and propensity score matching. These results suggest that treatment with erenumab earlier in the course of migraine may lead to a higher efficacy and clinical response in a real-world setting.

The European Headache Federation (EHF) updated its guideline in 2022, suggesting monoclonal antibodies targeting the CGRP pathway to be included as a first line treatment option for migraine patients requiring preventive treatment [23]. Still, most requirements for insurance coverage of CGRP targeted therapies include prior prophylactic medication attempts, with variations according to countries. For example, the National Institute for Health and Care Excellence (NICE) in the United Kingdom recommends at least three failures to preventive treatments [24–26]; in the United States, most managed care organizations require at least two or three previous attempts with alternative medications [27]; Canada’s Drug and Health Technology Agency (CADTH) recommends reimbursement if the patient had failed two or more medication attempts [28–30]. In Germany, for all CGRP mAbs other than erenumab, medication attempts with four prophylactic classes and onabotulintoxinA in case of chronic migraine are required. In some countries, like France, there is no reimbursement for CGRP (-R) mAbs and patients have to for them pay out of pocket [31].

One goal of requiring patients to try oral prophylactic medications first may be to save on medication costs, as some may be satisfied with a substantially cheaper medication. However, studies have shown that traditional “repurposed” oral prophylactic medications (antidepressants, beta blockers or anticonvulsants) have poor long-term adherence rates [3], with just 14% at 12 months in one study [32]. In another study from 2008, treatment persistence rates at day 360 after initiation of prophylactic medication was 4.8%, 8.7%, 6.1% and 8.8% for amitriptylin, beta blockers, valproate and topiramate respectively [33]. The most commonly cited reason for discontinuation was the occurrence of adverse events [34]. Treatment adherence to traditional oral prophylactic medications is unlikely to improve when patients know that alternative medications such as CGRP (-R) mAbs exist that are less likely to cause side effects and are possibly more effective. In many cases, the required medication attempts only postpone a therapy that is more costly but, in the end, might provide a substantial net benefit to society and patient alike. A recent pharmacoeconomic model from Denmark estimated that the initiation of CGRP (-R) mAbs results in a health economic savings of €1,179, €264, and €175 per year, and socioeconomic gains of €13,329, €10,449 and €9,947 per year for patients with chronic migraine (CM), high-frequency episodic migraine (HFEM) and low-frequency episodic migraine (LFEM) respectively, while the annual net cost of CGRP (-R) mAbs in Denmark is approximately €3,562 per year [35]. This would translate to a net benefit of €10,946, €7,151 and €6,560 for CM, HFEM and LEFM respectively. In another simulation, when treatment with erenumab was compared to best-supportive care for patients with at least four MMD and with two preventive treatment failures, treatment with erenumab resulted in a net benefit to society of €7,773 per patient [36].

Specifically for Germany, Seddik et al. calculated the potential value of CGRP inhibitors in a pharmacoeconomic simulation, if all eligible patients (i.e. all migraine patients with four or more MMD) received erenumab from 2020 to 2027. While it would cost the healthcare system an additional €8.4 billion per year, the sum of avoided loss of productivity through treatment and value chain effects would add up to €26.6 billion per year, leading to a net benefit for society of €18.2 billion per year [37].

Even more importantly, the results of our study imply that a postponement of CGRP targeted therapy could potentially lead to a decrease in its efficacy. In line with our observation, one study from Japan [38] describing a cohort with a mean 1.82 ± 0.11 prior prophylactic failures treated with either erenumab, fremanezumab or galcanezumab, reported a negative association between higher prior treatment failures and ≥ 50% response (OR with each additional prior prophylactic failure for ≥ 50% response in MMD at month 3: 0.512, 95% confidence interval: 0.290–0.904, *P* = 0.021). Another study using data from a compassionate use program of erenumab found higher 50% response rates for MHD at 3 months in patients that had two prior treatment failures than in those with more than two prior treatment failures (70% vs. 40.5%) [39]. A pooled analysis of real-world studies also showed that patients with a higher number of prior treatment failures were less likely to have a ≥ 50% response when treated with CGRP (-R) mAbs (odds ratio for ≥ 50% response in MHD or MMD: 0.83, 95% confidence interval: 0.73–0.93) [11].

Results from placebo-controlled trials suggest that a part of the increased efficacy with fewer prior preventives may be due to higher expectations and higher placebo effect in patients who have had fewer prior failures to preventive medications. Conversely, having repeated prophylactic attempts that end in failure may reduce the hope of success in further prophylactic attempts [40–42]. In our study, as it was not placebo-controlled, it is impossible to know what proportion of the better response in the new policy group is due to the placebo effect. The fact that the difference in response between old and new policy groups grew with increasing duration of therapy in our study, suggests that there is at least some contribution through true treatment response, as we would expect the placebo effect to decrease over time. Whether it was due to different expectations or true medication effect, starting CGRP (-R) mAbs earlier with the new coverage policy resulted in higher reductions in MHD, and higher ≥ 50% and ≥ 30% response rates, even after adjusting for baseline characteristics such as baseline headache frequency, disease duration or psychiatric comorbidities.

Our study has several limitations, first of which stems from the observational nature of the data, and the significant differences in baseline characteristics and size between the two cohorts being compared. However, to mitigate potential confounding, we have adjusted our analyses for baseline variables that differed between the two cohorts. A further limitation concerns the lack of a systematic collection of headache calendar in this real-world setting, which led to missing MMDs, and possible variations that led to a larger random error. Other outcomes such as quality of life, work productivity or depression, which may have provided additional insights, were not prospectively assessed.

Despite these limitations, our results suggest that starting erenumab earlier may lead to a significantly better treatment response than when erenumab is given as fifth or sixth-line therapy. Certainly, the choice of a prophylactic treatment for a given patient will remain an individual decision, taking into consideration various factors such as the patients’ comorbidities, the medications’ route and frequency of administration. Future studies might contribute to improved treatment-response prediction for different prophylactic treatments including oral preventatives, onabotulinomtoxinA and CGRP mAb as a step towards personalized migraine treatment, as recently shown for migraine acute medication [43].

## Conclusions

Results from our real-world observational study suggest that patients with migraine are likely to benefit more from treatment with erenumab if they are given access to it earlier, although confounding through baseline variables and the placebo effect cannot be excluded with certainty. If future studies confirm our results, this aspect of treatment with CGRP (-R) mAbs should be taken into account for a more accurate cost–benefit analysis to guide policy makers in their future decisions.

## Abbreviations

ATT: Average treatment effect of the treated
CGRP: Calcitonin gene-related peptide
CGRP (-R) mAb: Calcitonin gene-related peptide (-receptor) monoclonal antibody
CM: Chronic migraine
EHF: European Headache Federation
HFEM: High-frequency episodic migraine
IQR: Interquartile range
LFEM: Low-frequency episodic migraine
MHD: Monthly headache days
MMD: Monthly migraine days
PSM: Propensity score matching
RCT: Randomized clinical trial
SD: Standard deviation
SE: Standard error
SMD: Standardized mean difference
